# Editorial: Advances in microbial-based solutions: food coloring, flavoring and fragrance

**DOI:** 10.3389/fmicb.2026.1873693

**Published:** 2026-06-04

**Authors:** Laurent Dufossé, Rakesh Kumar, Bekzhan D. Kossalbayev

**Affiliations:** 1 CHEMBIOPRO Laboratory, Department of Food Science & Biotechnology, University of Réunion Island, Saint-Denis, France; 2 Department of Microbiology, CCS Haryana Agricultural University, Hisar, India; 3 Department of Chemical and Biological Technologies, Satbayev University, Almaty, Kazakhstan; 4 Department of Biotechnology, Faculty of Biology and Biotechnology, Al-Farabi Kazakh National University, Almaty, Kazakhstan

**Keywords:** bacteria, biotechnology, fungi, microorganisms, yeast

Microbial biotechnology is increasingly shaping the future of natural ingredients in the food, beverage, and fragrance sectors (Wang et al., 2026). Consumers and industries are seeking alternatives to synthetic additives and resource-intensive extraction from plants or animals, whereas producers require processes that are safe, scalable, reproducible, and economically viable. Bacteria (Sadanov et al., 2025), yeasts (Shurson, 2018), filamentous fungi (Maini Rekdal et al., 2024), and microalgae (Balouch et al., 2025) have a broad metabolic repertoire for producing pigments, aroma compounds, flavor precursors, enzymes, and stabilizing metabolites. Through strain selection, controlled fermentation, metabolic engineering, omics-guided process design, and circular use of agro-industrial by-products, microbial cell factories can help transform natural ingredient production into a more sustainable and technologically precise process (Li and Hall-Ponselè, 2025; Pullen et al., 2025; Verma et al., 2025).

This Research Topic brings together original research and review articles that illustrate the breadth of microbial-based solutions for coloring, flavoring, and fragrance. Together, these contributions highlight three connected directions: (1) the discovery and improvement of pigment-producing microorganisms, (2) the use of microbial consortia and starter cultures to shape flavor in fermented foods and beverages, and (3) the application of microbiome-guided strategies to improve aroma development in plant-derived products.

## Several contributions focused on microbial pigments and colorants

Karunarathna et al. reviewed Basidiomycete fungi as emerging sources of sustainable food colorants and stabilizers, emphasizing their pigmentation potential, biological activities and technological relevance. Noby et al. demonstrated a circular bioeconomy approach using cheese whey as a substrate for carotenoid production by *Agrococcus* sp. NP24, linking pigment production with waste valorization and bioactivity assessment. Duan et al. reported *P. lycopeni* A560 as a new lycopene-producing strain and optimized fermentation and extraction conditions, contributing to the development of microbial carotenoid production pipelines. These studies show that microbial pigments are moving from simple screening toward process optimization, substrate sustainability, compound recovery, stability, and application-oriented evaluation.

## A second group of contributions addressed flavor formation in fermented beverages

Caiza-Valencia et al. reviewed the role of native microorganisms as natural enhancers in craft beer and wine production, reinforcing the value of local microbial biodiversity for sensory complexity, authenticity, and sustainable innovation. Tang X. H. et al. screened high β-glucosidase-producing yeast strains from the Penglai wine region and evaluated their fermentation performance and aroma composition in Petit Manseng wine, illustrating how indigenous yeasts can release varietal aroma compounds from glycosidic precursors. Ruci et al. investigated Kallmet wine produced by sequential inoculation with *Metschnikowia pulcherrima* and *Saccharomyces cerevisiae*, demonstrating the potential of non-*Saccharomyces* yeasts to modulate wine quality. Piao et al. examined smoke-taint precursor modification by glycosidase activity in diverse wine yeast and bacterial strains, addressing an increasingly important challenge for winemaking in regions affected by wildfire smoke.

The importance of microbial ecology in traditional fermented products was particularly evident in studies on Baijiu focusing on physicochemical factors, microbial communities, and flavor compounds during the piling process of Furou-type Baijiu by Liu et al., while Tang Y. et al. analyzed microbial communities and flavor profiles in fermented grains of Furou-type Baijiu. Zeng et al. used multi-omics to show that glutinous rice varieties can shape Baijiu flavor through microbial and metabolic modulation. These studies illustrate how modern sequencing, metabolomics, and correlation analyses can decode complex solid-state fermentations and provide scientific support for improving traditional processes without altering product identity.

## Other contributions broadened the Research Topic toward fermented foods and aroma-rich plant materials

Holt et al. investigated solid-state Aspergillus oryzae koji fermentations of cereal and oilseed processing side streams, showing that substrate composition and moisture dynamics strongly shape fungal growth, physicochemical changes, and volatile organic compound formation. Their findings further demonstrate how fermentation stage, water activity, and substrate chemistry can guide aroma development while supporting the valorization of agro-industrial side streams. Sivamaruthi et al. reviewed the role of lactic acid bacteria in the meat industry, focusing on flavor development, functionality, and food safety. Shan et al. isolated indigenous *Bacillus velezensis* from aging tobacco leaves and demonstrated its potential for improving the flavor of flue-cured tobacco. Zhu et al. investigated microbial community succession during tobacco fermentation and revealed a flavor-improving mechanism involving functional microbial groups and targeted inoculation. Yan et al. further explored aroma-producing microorganisms from tobacco and evaluated their ability to enhance the flavor of naturally fermented essential oils. These articles reinforce the central idea of this Research Topic: microorganisms do not simply accompany fermentation, they actively structure aroma profiles through enzymatic conversion, precursor transformation, and metabolite production.

Several common themes emerged from the contributed papers in present Research Topic. First, microbial biodiversity remains a valuable reservoir for innovations in natural ingredient. Indigenous strains isolated from specific ecological or production environments often possess traits that are difficult to reproduce using generic industrial cultures. Second, omics technologies are essential for linking microbial taxa, functional genes, metabolites, and sensory outcomes. Third, process sustainability is no longer optional: the use of by-products such as cheese whey, the optimization of fermentation conditions, and the development of efficient extraction strategies are central to future industrial adoption.

The studies gathered in this Research Topic demonstrate that microbial biotechnology can contribute to safer, more sustainable, and more diverse natural ingredients for coloring, flavoring, and fragrances. Simultaneously, they showed that successful translation requires collaboration among microbiologists, food technologists, fermentation scientists, chemists, sensory scientists, regulatory specialists, and industry partners. The regulatory framework regarding the use of microbial food ingredients is complex and varies across the regions. The market will also play an important role as acceptance making consumer to understand myths on use of microbial driven food ingredients. Future research should continue to integrate strain discovery with systems biology, scale-up studies, machine learning, life-cycle thinking, and clear safety assessment. Such integration will be crucial for converting promising microbial metabolites into reliable commercial ingredients.

We thank all authors for their valuable contributions to this Research Topic, all reviewers for their constructive evaluations, and the Frontiers editorial team for their support throughout the editorial process. We hope that this Research Topic will stimulate further research and industrial innovation in microbial-based natural ingredients and will encourage the development of sustainable solutions for the food, beverage, and fragrance sectors.
